# The equivalence of measures on the Connectedness to Nature Scale: A comparison between ordinal methods of DIF detection

**DOI:** 10.1371/journal.pone.0207739

**Published:** 2018-11-19

**Authors:** Laura Pasca, María Teresa Coello, Juan Ignacio Aragonés, Cynthia McPherson Frantz

**Affiliations:** 1 University Complutense of Madrid, Madrid, Spain; 2 Oberlin College, Oberlin, Ohio, United States of America; Leibniz Institute for Research and Information in Education, GERMANY

## Abstract

The Connectedness to Nature Scale has been used in many different countries and settings. However, no one has yet tested the equivalence of these measures. Equivalence of measures has been the subject of much research in recent years, due to the importance of measuring in the same way when comparing between different groups. The present work studied the differential item functioning (DIF) of the CNS in a Spanish group and a North American group of respondents, using two different methods of detecting DIF. It also evaluated the overall equivalence of the scale. The results reveal differential functioning in most items, and only configural invariance is given. Thus, we suggest a reappraisal of the scale when comparing results from different countries since otherwise the conclusions drawn might be incorrect.

## Introduction

In Western culture nature plays an increasingly less important role in people’s existence. More and more people live in cities, and also spend more time in climate-controlled buildings rather than outdoors. However, nature continues to have an intrinsically positive value for people [1] and people empathize with both nature and the built environment [2].

Much research has been conducted in recent years on individuals’ connection to nature, which has in turn been linked to pro-environmental concern and behavior. These works can be grouped together under the perspective of environmental connectedness [3]. Relations between the self and nature are not only biophysical but can also be considered from the dimensions of cultural and spiritual evolution [4]. For these authors, these relations refer not only to how nature forms part of individuals’ identity, but also to how individuals perceive themselves as a part of nature.

Many methods for measuring nature connectedness exist (see [5] for a review). One of the most widely used is the *Connectedness to Nature Scale* (CNS) designed by Mayer and Frantz [6]. This scale consists of 13 items measured on a 5-point Likert-type scale. It has been translated into several languages and is currently used in many different parts of the world (e.g., [7, 8, 9, 10]). However, there are no studies showing that these versions and their applications to different groups are equivalent to each other. Do all individuals have the same understanding of connectedness to nature?

Equivalence of measures is a topic that has acquired a great deal of significance in research. In research based on multi-group comparisons, it is assumed that the instrument generally works in exactly the same way in different groups and that the construct of interest has the same structure. However, this is rarely demonstrated [11].

A test or scale presents equivalence (or invariance) of measures in various groups if respondents with the same score on the latent trait have the same expected score on the item, on the total test score, or on both [12, 13]. Equivalence of measures may also be understood as obtaining the same measurements of the same variables under different conditions, such as the passage of time, different populations, or the different methods used to administer the instrument [14]. The equivalence of measures is defined as [15]:

f(y|Θ,g)=f(y|Θ) (1)

where *y* is the set of measurements in a random sample of subjects, *g* indicates group membership, so that the set of observed scores, *y*, is the same for *g* if the probability of *y* given Θ, the latent variable, is the same regardless of group membership. An item (or test, or scale) is considered biased when members of a group are less likely to give a particular response than members of another group with the same score on the latent variable, because some aspects of the item that are not relevant to the purpose of the measure [16]. If there is no evidence of invariance of measurement, as occurs frequently [11], or if there is a lack of invariance, then there is no scientific basis for making inferences and the observed differences between groups or individuals cannot be clearly interpreted [17]. Observed differences might be due to a true difference in the variable, a difference in the relationship between the construct and the observed variable, or some combination of both [18]. The patterns of correlations with other variables may also be erroneous [13].

The validity of comparisons of scores obtained in different countries or in different cultures is vital in applied and cultural psychology [19]. Differential item functioning is a serious threat to test validity [20, 21], since such comparisons are based on the premise of construct comparability, which necessitates that the test (or scale) scores from different countries measure the same construct on the same metric [22].

Quantitative assessment of equivalence has been approached in a variety of ways. Most research on *Differential Item Functioning* (DIF) has been developed for dichotomous items. Nevertheless, the use of polytomous items is very common in psychological research [23], including the most common measures of connectedness to nature. In dichotomous response items, DIF exists when the probability for a correct response, given the same score on the latent variable, differs for different groups, while, in polytomous items, defining DIF is more complex since this can remain constant or differ in the different response categories [24].

The use of polytomous items requires reconsidering some of the psychometric procedures created specifically for dichotomous items [25]. In particular, Spray and Miller, point out that the identification of differential item functioning within each of the categories of a polytomous item requires either modifications of the procedures used for dichotomous items, or the creation of new procedures suitable for this type of items. In order to respond to this need, studies have been developed comparing and testing different methods of detecting differential functioning in ordinal items (e.g., [26, 27]).

There are two types of procedures for the detection of differential functioning; those based on the observed score and those based on the latent variable [28]. The latter methods include models of *Item Response Theory (IRT)* and models of *Confirmatory Factor Analysis* (CFA), while the former include methods based on contingency tables, such as that designed by Mantel-Haenszel (MH), and those based on the adjustment of nested Logistic Regression (LR) models. An important difference between the four procedures outlined is that those based on IRT and CFA models require the specification of a measure model, in addition to a sufficient sample size to permit estimation of the parameters. Neither LR (based on the adjustment of nested models), nor MH (based on contingency tables) [23] present this requirement. Although these methods share some common characteristics [29], broadly speaking, there is a tendency to carry out an isolated use of one or the other method, drawing on different approaches that also imply procedures and estimation of different parameters. This analysis uses both.

The major aim of the present work is to determine whether there exists an equivalence of measures in the CNS between two groups, one from the United States and one from Spain. A second aim is to compare different methods in the detection of differential functioning in empirical samples, since most of the previous comparisons have been conducted in simulated samples (e.g., [23, 24]). Specifically, DIF analysis will be carried out using MH and LR, and CFA will be used to analyze the scale as a whole (this analysis excludes IRT models because they require larger groups than those available).

The first of the methods, the Generalized Mantel-Haenszel test [30], draws on the degree of association in contingency tables to test the null hypothesis of non-association between response and group, controlling for the effect of the covariate (the total score of the scale). The second method, LR [31, 32, 33, 34] models a relation between a criterion or response variable and a set of predictor variables. The response variable is the item score, while the predictor variables are group, total test score and the interaction between both variables. The fit of a series of nested models from the most complex to the simplest allows to determine the presence/absence of DIF. Finally, CFA [35] models the relations between the observable measures or indicators and the latent variables or factors. The invariance of the measures is established by testing a hierarchical series of models with increasing restrictions. A significant decrease in fit between the models indicates differences between the groups in the most restricted matrix [36]. There are three models needed to confirm factorial invariance. The first model tests the configural invariance, that is, whether people from different groups have the same concept of the construct. The second one checks the metric invariance, if, in addition to the above, the strengths of the relations between each item and the underlying construct are the same across groups. The third model tests the scalar invariance, namely, if the intercepts are the same across groups, showing that observed scores are the same across groups when the latent variable is identical.

It is hypothesized that there is no differential item functioning between the American and Spanish groups, and that, therefore, the two versions are equivalent.

## Method

### Participants

As the reference group, we used a sample of 361 American individuals with a mean age of 31.29 years (SD = 17.06). This group was taken from the studies by Mayer and Frantz, authors of the original CNS [6]. Four databases were used to form this sample, two comprised of students and two comprised of adult samples.

As a comparison group, we used data from different studies in which the Connectedness to Nature scale was administered in Spanish to students of psychology [37, 38, 39, 40, 7, 8]. By unifying these databases, a total group of 1504 participants was obtained, of which 1153 were women and 351 were men, with a mean age of 23.15 years (SD = 7.45).

To match the sample size of both groups, a random sample of 384 cases from the Spanish group was selected. Therefore, the total sample used in the comparison of the groups consisted of 745 cases.

### Instrument

We used the CNS [6], which consists of 13 Likert-type items, in which participants must position themselves on a continuum of five points. In the present work, as well as the original scale in English, the adaptation to Spanish [37] was used for the Spanish sample.

### Design

The study design is a cross-sectional survey, which allows to collect the information on the variables of interest through the CNS. The two groups used, Spanish and North American, are incidental samples, meaning they have been selected because of their availability.

### Data analysis

First, to test the mean equality hypothesis between the Spanish group and the American group, a Mann-Whitney U test for independent samples was performed. Subsequently, the dimensionality of the scale was analyzed, using the FACTOR 9.3 program [41].

In order to meet the aim of detecting the differential functioning in the items and to verify the hypothesis of the present work, three procedures were used. The first is the generalized Mantel-Haenszel statistic for ordinal response variables, QMH (2), which allows to contrast the null hypothesis of non-association between variables, being rejected if the value of the statistic *P*(*Q*_*MH*(2)_) < 0.05. The rejection of the null hypothesis for an item implies it exhibits DIF. This analysis was conducted using the GMHDIF program [42], use of which was granted by its author.

Logistic regression was conducted using proportional reasoning on the items that complied with the parallel lines assumption, and partial proportional reasoning for the remaining items. We followed the criterion of Swaminathan and Rogers [43], comparing the fit of the different nested models with the Probability Ratio (RV) statistic, which follows the distribution $\chi_{\left(1\right)}^{2}$ and a significance level of .005 ($\chi_{\left(1\right)}^{2}=7.88)$. These analyses were conducted using the SAS 9.4 package.

Finally, to determine whether the questionnaire is equivalent in the two languages, the invariance of the test in English and Spanish was analyzed, examining three aspects: 1) dimensionality; 2) measurement pattern and 3) error variance. This analysis was implemented using the LISREL 8 program [44].

The level of significance used in the analyses was .05. Bonferroni correction was used in the DIF analysis.

## Results

### Mean and variance

The homogeneity hypothesis of the variances of the total score of the scale was tested in both groups (American and Spanish), using the F test. The results lead us to reject the homogeneity hypothesis (F (1.743) = 12.81, p < .005).

The Mann-Whitney U test for independent samples, to test the equality hypothesis of the total mean scores on the scale was not statistically significant (Z = -.392, p > .05). Thus, it was concluded that the US and Spanish averages are not statistically different, that is, both groups have the same average score in Connectedness to Nature.

### Dimensionality analysis

The CNS was created as a unidimensional measure. However, some authors have indicated that more than one component could be measured (e.g. [45]). Consequently, before studying the differential functioning, a parallel analysis was conducted, in order to verify the number of factors. In both cases, when analyzing the polychoric correlation matrix, the parallel analysis suggests the existence of one single factor that explains 31.8% (KMO = .846) of variance in the Spanish group, and 46% (KMO = .89) in the American group.

### DIF analysis

#### Generalized Mantel Haenszel Method

In the differential functioning analysis of the items using the generalized Mantel Haenszel method (MH), we used four strata formed from participants’ total scores on the scale [42]. The first stratum covered the range from 18 to 30 points, the second from 31 to 43, the third from 44 to 56, and the last from 57 to 70. The results of the analysis for the two stages are shown in Table 1.

**Table 1:** DIF analysis using the generalized Mantel Haenszel Method.

	Step 1			Step 2		
Item	Q_GMH(2)_(χ^2^)	Df	Sig.	Q_GMH(2)_(χ^2^)	df	Sig.
1	0.515	1	0.473	0.001	1	0.975
2	3.990	1	0.046	6.116	1	0.013
3	3.990	1	0.046	3.065	1	0.080
4	22.313	1	0.000*	20.382	1	0.000*
5	17.277	1	0.000*	15.013	1	0.000*
6	39.809	1	0.000*	38.992	1	0.000*
7	0.375	1	0.540	0.855	1	0.355
8	69.913	1	0.000*	54.464	1	0.000*
9	43.902	1	0.000*	33.733	1	0.000*
10	4.417	1	0.036	6.776	1	0.009
11	8.598	1	0.003*	7.994	1	0.005*
12	12.273	1	0.001*	12.874	1	0.000*
13	1.089	1	0.297	2.285	1	0.131

*p<0.005

Applying the Bonferroni correction, items 4, 5, 6, 8, 9, 11 and 12 present differential functioning between the two languages (see Table 1). That is, 61.54% of the items that make up the scale do not measure connectedness in the same way in each group.

However, if we took a significance level of .05, the number of items with DIF would increase to 9.

#### Ordinal logistic regression

In differential item functioning analysis using ordinal logistic regression (OLR), three models were fitted, from the most parsimonious to the most complex. Model 1 includes a single explanatory variable, the total score on the scale. Model 2 includes, in addition to the total score, the group effect (Spanish and American). Finally, model 3 also includes the interaction effect between both coefficients.

Prior to examining the fit of the models, compliance with the proportionality (parallelism) assumption was analyzed, since, depending on compliance, a proportional or non-proportional odds model should be adjusted. Only items 3, 6 and 8 complied with this assumption. Thus, for the remaining items a partial proportional odds model was fitted. After this verification, DIF analysis was performed, comparing the three models for each item.

Results are shown in Table 2.

**Table 2:** DIF analysis by ordinal logistic regression.

Item	Model	RV (χ^2^)	df	Difference in RV (χ^2^)	Dif. df
1	M1	407.706	4		
	M2	408.216	5	0.510	1
	M3	415.441	9	7.224	4
2	M1	425.968	1		
	M2	446.896	5	20.927****	4
	M3	449.566	9	2.670	4
3	M1	264.568	1		
	M2	268.101	2	3.533	1
	M3	268.891	3	0.790	1
4	M1	224.168	4		
	M2	249.037	5	24.869****	3
	M3	254.048	9	5.011	4
5	M1	294.666	1		
	M2	339.225	5	44.559****	4
	M3	342.922	9	3.696	4
6	M1	377.345	1		
	M2	426.616	2	49.271****	1
	M3	427.122	3	0.506	1
7	M1	319.328	1		
	M2	341.165	5	21.837****	4
	M3	363.882	9	22.717****	4
8	M1	194.534	1		
	M2	273.047	2	78.513****	1
	M3	275.278	3	2.231	1
9	M1	426.567	4		
	M2	476.046	5	49.479****	3
	M3	482.493	9	6.447	4
10	M1	389.938	4		
	M2	428.847	8	38.910****	4
	M3	440.871	12	12.023*	4
11	M1	480.971	4		
	M2	491.066	5	10.095*	3
	M3	501.552	9	10.486*	4
12	M1	147.312	1		
	M2	180.487	5	33.175****	4
	M3	190.054	9	9.568*	4
13	M1	162.549	1		
	M2	188.751	5	26.202****	4
	M3	191.414	9	2.663	4

*p<0.05;

****p<0.001

From the data in Table 2, it can be seen that items 1 and 3 are the only ones not exhibiting differential functioning, since there is no group effect. On the other hand, items 2, 4, 5, 6, 8, 9, 10, 12 and 13 present uniform DIF, that is, differential operation occurs in the same way in individuals with different levels of connectedness. Item 7, however, presents non-uniform DIF, that is, differential functioning differs according to individuals’ level of connectedness.

#### Confirmatory Factor Analysis (CFA)

To complete the above analyses, and in order to verify whether measurement equivalence can be established for the scale as a whole, a multi-group CFA was carried out.

The same configuration was seen to exist in both groups (Table 3), having an acceptable fit to the data (χ^2^ (130) = 379.07; RMSEA = 0.05; CFI = 0.89), so we proceeded with the analysis of metric invariance, that is, the equality of factorial weights. When fitting the model in which the equality hypothesis of factorial weights is compared across the both groups, the ΔRSMEA and ΔCFI are less than 0.01, and a value of χ^2^ (143) = 436.06 are obtained. However, if the Chi-Square statistics of both models are compared -the first model without restrictions in the weights and the second in which equality is required-, a statistically significant value of Δχ^2^(13) = 56.99 (p < .05) is obtained, and Δχ^2^/df is higher than 3, so the invariance of the measures cannot be assumed. In other words, the factorial weights differ between groups.

**Table 3:** RMSEA, CFI, χ^2^ and χ^2^/df in the different invariance models.

	Configural invariance		Metric invariance		Scalar invariance	
CNS	RMSEA	.05	ΔRMSEA	.00	ΔRMSEA	.00
	CFI	.89	ΔCFI	-.01	ΔCFI	-.01
	χ^2^	379.07	Δχ^2^	56.99*		
	χ^2^/df	2.91	Δχ^2^/df	4.38		

*p < .05

After determining there was no equivalence in the measures, it was impossible to continue the study process imposing other restrictions.

## Discussion

The study of measure equivalence is of great practical utility, since scales and tests are continually administered to very diverse groups of individuals. Indeed, it is often necessary to translate the instruments, as is the case with the CNS by Mayer and Frantz [6]. Nevertheless, the efforts to guarantee the equivalence of the instruments may be ineffective, since the items are not always understood in a similar way in all cultures, and the use of the scale may be conditioned by the cultural context [19]. When using a scale on individuals with different characteristics, it is assumed that those with the same observed score on the scale would have the same level on the instrument’s underlying construct [46]. However, the results found in this work show that this does not occur in the Connectedness to Nature scale, at least among the two groups studied here. This means that individuals with the same degree of connectedness might yield different scores on the scale, or those with the same scores might have varying degrees of connectedness.

Using the generalized Mantel-Haenszel method, we found that 7 of the 13 items comprising the scale show differential functioning. However, this statistic does not allow us to distinguish whether it is uniform or not. This number is high, accounting for more than half the items on the scale. Therefore, according to the results obtained from this statistic, it would be necessary to reduce the scale to 6 items for an adequate comparison between the data obtained on the scale applied to the American sample and that applied to the Spanish sample. However, reliability would decrease from an alpha of .811 to an alpha of .672.

The analyses conducted using the LR method show that 11 of the 13 items present uniform differential functioning between the samples, which means that the probability of answering a determined category is greater for one group than the other across all trait levels. None of the items present non-uniform differential functioning.

Nonetheless, despite the items exhibiting differential functioning, there might exist some compensation across items at scale level, resulting in equivalence of measures when applying the scale as a whole. To verify this, we analyzed invariance using CFA. The levels of the invariance analysis show that, although both groups present the same configuration, it cannot be assumed that the factorial weights are the same, that is, there is no metric invariance. Therefore, after comparing the equivalence of the measures from the three previous methods, it can be concluded that the results obtained on the CNS in the American (CNS in English) and Spanish (CNS in Spanish) samples are not comparable, since the versions are not equivalent. Consequently, this instrument cannot be used for comparative studies between these two groups, since a particular score in one group is not necessarily equivalent to the same score in the other group. Nevertheless, the scale can still be used, provided groups are not compared with each other. It is possible that invariance of measures could be found between a different pair of groups using the same scales. However, this could only be determined by analyzing the equivalence of measurements.

This approach supports the results obtained by Davidov and De Beuckelaer [47] who, following a cross-cultural study, suggested that translations of scales and tests may seriously distort the comparability of results between different countries. This is not necessarily the result of a poor translation, but rather of cultural differences when it comes to understanding a particular construct, or different use of language, since certain words might be commonly used in some cultures but unusual in others.

Construct bias is the most common form of bias, denoting that the underlying theoretical concept itself has a different meaning for different groups [19]. As these authors indicate, a good translation could avoid some of the bias in the items, although this does not imply that in different samples the concept is understood in the same way. In relation to the construct analyzed in this study, some works show cultural differences when it comes to understanding nature (e.g., [48]). Hence, if nature does not mean the same thing in different cultures, it is to be expected that connectedness cannot be measured in the same way.

By measuring connectedness in different contexts, the meaning of connectedness could be different and, consequently, an assessment of the scale would be necessary, that is, carry out an equalization of scores in the different countries. For this, a new study would have to be carried out, in which different scores obtained in both groups were compared, for which large samples from different countries would be needed.

Moreover, the characteristics of the samples used should be taken into account. The data of the American group was published in 2004, while the Spanish data was taken from studies published on different dates up to 2014. The conception of nature and connectedness is different in different spatial and/or cultural contexts, as has been shown in this paper, but it may also change over time. Thus, it would be advisable to study whether differences in the concept arose for the same groups at different moments in time.

Therefore, it would be desirable that whenever the results obtained in different countries are compared, they should be interpreted with caution, since it is not possible to decide in this work whether the differences are attributable to the different social context or to the different moment of data collection, or to an interaction of both aspects.

## Supporting information

S1 Data Anonymized data CNS. (XLSX)
